# Mortality risk from acute aortic dissection among hospital admissions during weekends and holiday season

**DOI:** 10.1371/journal.pone.0255942

**Published:** 2021-09-01

**Authors:** I-Min Su, Huei-Kai Huang, Peter Pin-Sung Liu, Jin-Yi Hsu, Shu-Man Lin, Ching-Hui Loh

**Affiliations:** 1 Department of Anesthesiology, Hualien Tzu Chi Hospital, Buddhist Tzu Chi Medical Foundation, Hualien, Taiwan; 2 Departments of Family Medicine and Medical Research, Hualien Tzu Chi Hospital, Buddhist Tzu Chi Medical Foundation, Hualien, Taiwan; 3 School of Medicine, Tzu Chi University, Hualien, Taiwan; 4 Center for Aging and Health, Hualien Tzu Chi Hospital, Buddhist Tzu Chi Medical Foundation, Hualien, Taiwan; 5 Institute of Medical Sciences, Tzu Chi University, Hualien, Taiwan; 6 Department of Physical Medicine and Rehabilitation, Hualien Tzu Chi Hospital, Buddhist Tzu Chi Medical Foundation, Hualien, Taiwan; Kaohsuing Medical University Hospital, TAIWAN

## Abstract

**Background:**

Acute aortic dissection is a life-threatening condition associated with high mortality rate. Findings from previous studies addressing the “weekend effect” on the mortality rate from an acute aortic dissection mortality have been inconsistent. Furthermore, the effect of admission for acute aortic dissection during the holiday season has not been previously investigated.

**Objective:**

Our aim was to evaluate the effect of admission for acute aortic dissection during holiday season or weekends on the risk of mortality.

**Methods:**

We conducted a retrospective analysis of nationwide cohort data from the Taiwan’s National Health Insurance Research Database. We collected data on all adult patients hospitalized for acute aortic dissection between 2001 and 2017 in Taiwan and classified them into the following three groups based on day of admission: holiday season (at least 4 consecutive days; n = 280), weekend (n = 1 041), and weekday (n = 3 109). The following three outcomes were evaluated: in-hospital mortality, 7-day mortality, and 180-day mortality.

**Results:**

A multivariable logistic regression was used to adjust for possible cofounders on the measured outcomes. Compared to weekday admissions for acute aortic dissection, weekend admissions resulted in a 29% increase in the risk of in-hospital death (aOR = 1.29; 95% CI, 1.05–1.59; P = 0.0153), with a 25% increase in the 7-day (aOR = 1.25; 95% CI, 1.001–1.563; P = 0.0492) and 20% increase in the 180-day mortality risk (aOR = 1.20; 95% CI, 1.01–1.42; P = 0.0395). Of note, admission over the holiday season did not result in a higher mortality risk than for weekday admissions; this finding, however, might reflect insufficient statistical power on subgroup analysis.

**Conclusion:**

Patients admitted for acute aortic dissection during the weekends are at higher risk of mortality compared to those admitted on weekdays. Our finding likely reflects inadequate staffing and team experience of weekend staff and can guide healthcare policy makers to improve patient outcomes.

## Introduction

Acute aortic dissection is a life-threatening condition associated with high mortality rate. Despite recent advances in diagnostic and treatment strategies, in-hospital mortality rate was as high as 22% and 14% for patients with acute type A and type B aortic dissection, respectively, according to a recent analysis from the International Registry of Acute Aortic Dissection [1]. Accordingly, this condition requires immediate medical intervention.

Patients with acute aortic dissection may present to a hospital at any time, including nights, weekends, and holidays, requiring emergency surgery or admission to intensive care. Within hospitals, having adequate staffing and clinical experience are key factors for effective management of this critical disease and, thus, enhance survivability. However, in the real world, shortage of staff [2] and lack of staff with subspecialty training [3] during weekends results in a higher risk of mortality for patients admitted with acute aortic dissection, a phenomenon known as the “weekend effect” [4, 5]. Nevertheless, the magnitude of this effect remains undetermined. A recent analysis from a Nordic Aortic Dissection Database, however, observed no significant relationship between weekend admissions and mortality risk from an acute aortic dissection [6].

In Taiwan, the Chinese New Year is celebrated annually every January or February and includes at least four consecutive days of holidays. During such holiday periods, staffing level in hospitals is remarkably lower; the diminished availability of medical personnel and resources is a more serious problem than those on weekends. Yet, to date, the possible effect of admission over a holiday period, such as the Chinese New Year, on the mortality risk for acute aortic dissection has not been evaluated. We conducted a nationwide study to evaluate the potential effect of “weekend” and “holiday season” admissions on the mortality risk for acute aortic dissection in a Taiwanese cohort.

## Materials and methods

### Data sources

This was a retrospective analysis of the clinical and mortality data available in Taiwan’s National Health Insurance Research Database (NHIRD) for patients admitted with a diagnosis of acute aortic dissection. Taiwan’s National Health Insurance (NHI) is a compulsory social health program managed by the government that covers 99% of Taiwan’s population. The NHI program, implemented in 1995, has contracts with 97% of the clinics and hospitals in Taiwan. Thus, the NHIRD data is sufficient to represent the entire population of Taiwan, containing detailed medical records for about 25 million individuals [7]. The NHIRD provides high quality information suitable for research including diagnosis, hospitalization, surgical history, and prescription use. Access to these data for research is provided by permission from the Health and Welfare Data Science Center in Taiwan. Before 2016, the diagnostic and procedure codes were based on the International Classification of Diseases, Ninth Revision, Clinical Modification (ICD-9-CM); since 2016, the International Classification of Diseases, Tenth Revision, Clinical Modification (ICD-10-CM) has been used for these purposes. The study protocol was approved by the Hualien Tzu Chi Hospital Research Ethics Committee (IRB107-152-C), with the requirement for informed consent waived owing to the retrospective design of the study.

### Study cohort

We extracted the data for all patients >20 years of age with a new diagnosis of acute aortic dissection over the study period, namely January and February for each year between 2001 and 2017. The diagnosis of acute aortic dissection was identified using the ICD-9-CM codes (441.0, 441.00, 441.01, 441.02, and 441.03) and the ICD-10-CM codes (I71.0, I71.00, I71.01, I71.02, and I71.03). According to Stanford system definitions, dissections that involve the ascending aorta are type A, regardless of the site of the primary intimal tear. All other dissections are defined as type B [8]. To isolate Type A from Type B aortic dissection, we identified patients based on the following procedure code in both the ICD-9-CM Procedure Code (38.34 and 38.44) and ICD-10-CM Procedure Code (02QX and 02QRX) and the hospitalized surgery codes in NHIRD (68043B and 69035B), which are most likely applicable to Type A aortic dissection.

According to the admission date, patients were classified into the following three admission groups: weekday, weekend, and holiday season. We chose the Chinese New Year holiday, which typically includes at least 4 days per year, as a representative holiday season period. As this period holiday can be extended if it coincides with a weekend, the exact dates of the Chinese New Year holidays in each year were confirmed using Taiwan’s national public holiday list. The weekday group was defined as admissions between Monday and Friday and the weekend group as admissions on Saturday and Sunday; patients admitted during the Chinese New Year holidays were excluded from these two groups. Since the Chinese New Year holiday is always in January or February, to control for possible seasonal effects on acute aortic dissection [9], we only included patients admitted during the months of January and February, annually, as our study population. As our target population were patients with a newly diagnosed aortic dissection, patients with any previous diagnosis of aortic dissection made either on an inpatient or outpatient basis before the index date of admission were excluded. Patients who were hospitalized for more than 180 days during the index hospitalization were also excluded to eliminate possible outliers.

### Outcome measures

The date of admission for acute aortic dissection was defined as the index date; the corresponding hospitalization was defined as the index hospitalization. To ensure the integrity of mortality outcomes, we linked data from Taiwan’s National Register of Deaths to the NHIRD data. Our primary outcomes were in-hospital mortality and mortality within 7 days and 180 days (for evaluating the short- and long-term outcomes, respectively) after admission. We defined in-hospital mortality as death during index hospitalization (namely, discharge by death). The 7-day mortality was defined as any death occurring within 7 days after the index date; the 180-day mortality was defined as any death occurring within 180 days after the index date. The choice of the 7-day and 180-day mortality for evaluating short- and long-term prognosis was based on previous studies as references [10, 11]. Data from patients admitted on a weekday were used as the reference to compare the mortality risk for weekend and holiday season admissions. Subgroup analyses were conducted after stratification for sex, age, acute aortic dissection type, and hospital area.

### Covariates

Patient baseline characteristics, including demographic data, comorbidities, and clinical details were obtained from inpatient, outpatient, and emergency service reimbursement claims, using the ICD-9-CM, ICD-10-CM, and procedure codes. Pre-existing comorbidities were included when they were diagnosed at least once in the inpatient clinic or twice in the outpatient clinic within a year prior to the index date of admission. The Charlson comorbidity index was also calculated according to each patient’s medical records. The Charlson comorbidity index is widely used to represent an individual’s overall systemic health status and is highly correlated with mortality risk [12]. Income was estimated from the income related NHI premiums and categorized into four levels (New Taiwan dollars ≥40 000, 25 000–39 999, 15840–24999, and financially dependent [<15 840]). Hospitals where patients were admitted were classified into three levels according to their accreditation level (medical center, regional hospital, or district hospital). In Taiwan, hospital level is accredited by the Taiwan Joint Commission on Hospital Accreditation based on clinical capabilities, healthcare qualities, bed capacities, and medical teaching abilities [13]. The hospital area was classified as North, Central, South, and East of Taiwan.

### Statistical analysis

A t-test was used to analyze continuous variables and a chi-squared (χ^2^) test for categorical variables. We calculated the odds ratio (OR) and the respective 95% confidence interval (CI) for in-hospital, 7-day, and 180-day mortality using logistic regression models. Multivariable logistic regression model was performed, with adjustment for all covariates listed in Table 1 (including age, sex, income level, aortic dissection type, hospital level, hospital area, Charlson comorbidity index score, and comorbidities). In addition, we performed a sensitivity analysis applying a multivariable Cox proportional hazards regression model to estimate the adjusted hazard ratio (aHR), which was assumed to be constant over time. In the Cox regression model, we followed up patients from the index date (date of admission for acute aortic dissection) until death or the 180^th^ day after the index date (follow-up for 6 months). Interaction tests were performed to determine whether the subgroup factors (sex, age, acute aortic dissection type, and admission hospital area) were potential effect modifiers. A two-sided probability value <0.05 was considered statistically significant. Statistical analyses were performed using SAS (version 9.4, SAS Institute, Inc., Cary, NC).

**Table 1:** Baseline characteristics of patients admitted during the holiday season, weekend, and weekdays.

	Holiday Season		Weekend		Weekday		p for comparison between groups		
	n = 280		n = 1041		n = 3109		Holiday season vs. Weekday	Weekend vs. Weekday	Holiday season vs. Weekend
	n	%	n	%	n	%			
Age (years)*	65.8 ± 14.0		64.8 ± 14.6		65.3 ± 14.8		0.6131	0.3105	0.3044
Age categories (years)							0.8839	0.0685	0.7467
<40	10	3.6	37	3.6	151	4.9			
40–49	30	10.7	135	13	313	10.1			
50–59	54	19.3	216	20.8	638	20.5			
60–69	65	23.2	240	23.1	709	22.8			
70–79	69	24.6	216	20.8	702	22.6			
≥ 80	52	18.6	197	18.9	596	19.2			
Sex							0.5141	0.3275	0.2569
Male	189	67.5	739	71.0	2157	69.4			
Female	91	32.5	302	29.0	952	30.6			
Income level (New Taiwan Dollars)							0.0040	0.2184	0.0004
Financially dependent	25	8.9	134	12.9	364	11.7			
15 840–24 999	125	44.6	325	31.2	1061	34.1			
25 000–39 999	73	26.1	320	30.7	883	28.4			
≥40 000	57	20.4	262	25.2	801	25.8			
Aortic dissection type							0.5729	0.0976	0.1850
Type B	267	95.4	970	93.2	2940	94.6			
Type A	13	4.6	71	6.8	169	5.4			
Hospital level							0.0161	0.0351	0.3879
Medical center	187	66.8	649	62.3	1803	58.0			
Regional hospital	84	30.0	356	34.2	1168	37.6			
District hospital	9	3.2	36	3.5	138	4.4			
Hospital area							0.0618	0.6156	0.0884
North	98	35.0	441	42.4	1267	40.8			
Central	78	27.9	230	22.1	665	21.4			
South	92	32.9	334	32.1	1061	34.1			
East	12	4.3	36	3.5	116	3.7			
Charlson comorbidity index score							0.2665	0.0361	0.2674
0	171	61.1	639	61.4	1780	57.3			
1–2	72	25.7	297	28.5	944	30.4			
≥3	37	13.2	105	10.1	385	12.4			
Comorbidities									
Diabetes mellitus	32	11.4	117	11.2	317	10.2	0.5158	0.3412	0.9291
Hypertension	148	52.9	524	50.3	1580	50.8	0.5137	0.7869	0.4538
COPD	28	10.0	83	8.0	315	10.1	0.9441	0.0406	0.2778
Heart failure	17	6.1	59	5.7	220	7.1	0.5278	0.1162	0.7967
Coronary artery disease	41	14.6	188	18.1	587	18.9	0.0805	0.5563	0.1800
Chronic kidney disease	23	8.2	70	6.7	213	6.9	0.3907	0.8883	0.3870
Cirrhosis	17	6.1	49	4.7	165	5.3	0.5869	0.4485	0.3522
Stroke	26	9.3	106	10.2	336	10.8	0.4298	0.5717	0.6569
Dementia	9	3.2	32	3.1	72	2.3	0.3458	0.1756	0.9043
Malignancy	11	3.9	43	4.1	168	5.4	0.2905	0.1056	0.8795

Continuous variables are compared using t-tests; categorical variables are compared using χ2 tests.

*Expressed as mean ± SD.

Abbreviations: n, number; SD, standard deviation; COPD, chronic obstructive pulmonary disease.

## Results

### Patient characteristics

There were 4430 admissions for acute aortic dissection in the months of January and February between 2001 and 2017. Of these, 3109 admissions were on weekdays, 1041 on weekends, and 280 during holiday season. The mean age of study individuals was 65.8, 64.8, and 65.3 years in holiday season, weekend, and weekday groups, respectively. The proportion of men was higher than that of women in all the three study groups. Most of the patients were admitted to medical centers (the highest hospital level in Taiwan). Hypertension, coronary artery disease, and diabetes mellitus were the top three prevalent comorbidities in the study population. Overall, the among-group differences were minimal in most of the baseline characteristics, except income level and hospital level (Table 1).

### Mortality risks

Using the logistic regression model adjusted for the relevant characteristics listed in Table 1, a weekend admission was associated with a 29% increased risk of in-hospital mortality compared to a weekday admission (adjusted odds ratio [aOR] = 1.29; 95% confidence interval [CI], 1.05–1.59; P = 0.0153), a 25% increase in the 7-day mortality (aOR = 1.25; 95% CI, 1.001–1.563; P = 0.0492), and a 20% increase in the 180-day mortality (aOR = 1.20; 95% CI, 1.01–1.42; P = 0.0395) (Table 2). Although a similar pattern of higher risk on all three mortality outcomes was identified for admission during the holiday season compared to a weekday admission, differences were not significant: in-hospital mortality (aOR = 1.19; 95% CI, 0.83–1.71; P = 0.3452); 7-day mortality (aOR = 1.13; 95% CI, 0.77–1.68; P = 0.5315), and 180-day mortality (aOR = 1.06; 95% CI, 0.79–1.43; P = 0.6886) (Table 2).

**Table 2:** Mortality risks for the patients admitted for acute aortic dissection during the holiday season, weekends, and weekdays.

Outcome	Day type	N	Mortality		Univariable model			Multivariable model		
			n	%	OR	95% CI	p-value	aOR*	95% CI	p-value
In-hospital mortality	Holiday season	280	38	13.6	1.21	0.85–1.74	0.2896	1.19	0.83–1.71	0.3452
	Weekend	1041	149	14.3	1.29	1.05–1.59	0.0147	1.29	1.05–1.59	0.0153
	Weekday	3109	356	11.5	1	Ref	Ref	1	Ref	Ref
7-day mortality	Holiday season	280	32	11.4	1.14	0.77–1.67	0.5160	1.13	0.77–1.68	0.5315
	Weekend	1041	126	12.1	1.21	0.97–1.51	0.0848	1.25	1.001–1.563	0.0492
	Weekday	3109	317	10.2	1	Ref	Ref	1	Ref	Ref
180-day mortality	Holiday season	280	66	23.6	1.08	0.81–1.45	0.5872	1.06	0.79–1.43	0.6886
	Weekend	1041	255	24.5	1.14	0.97–1.34	0.1201	1.20	1.01–1.42	0.0395
	Weekday	3109	689	22.2	1	Ref	Ref	1	Ref	Ref

*The aOR is calculated by multivariable logistic regression model with adjustments for the characteristics listed in Table 1 (including age, sex, income level, aortic dissection type, hospital level, hospital area, Charlson comorbidity index score, and comorbidities).

Abbreviations: n, number; OR, odds ratio; aOR, adjusted odds ratio; CI, confidence interval; Ref, reference group

There was no significant difference in mortality outcomes between holiday season and weekend admissions: in-hospital (aOR = 0.87; 95% CI, 0.59–1.30; P = 0.5046), 7-day (aOR = 0.89; 95% CI, 0.58–1.36; P = 0.5837), and 180-day (aOR = 0.87; 95% CI, 0.63–1.21; P = 0.4085) mortality (Table 3).

**Table 3:** Mortality risks between patients admitted for acute aortic dissection during the holiday season and those admitted on weekends.

Outcome	Day type	N	Mortality		Univariable model			Multivariable model		
			n	(%)	OR	95% CI	p-value	aOR*	95% CI	p-value
In-hospital mortality	Holiday season	280	38	13.6	0.94	0.64–1.38	0.7520	0.87	0.59–1.30	0.5046
	Weekend	1041	149	14.3	1	Ref	Ref	1	Ref	Ref
7-day mortality	Holiday season	280	32	11.4	0.94	0.62–1.42	0.7573	0.89	0.58–1.36	0.5837
	Weekend	1041	126	12.1	1		Ref	1		Ref
180-day mortality	Holiday season	280	66	23.6	0.95	0.70–1.30	0.7489	0.87	0.63–1.21	0.4085
	Weekend	1041	255	24.5	1	Ref	Ref	1	Ref	Ref

*The aOR is calculated by multivariable logistic regression model with adjustments for the characteristics listed in Table 1 (including age, sex, income level, aortic dissection type, hospital level, hospital area, Charlson comorbidity index score and comorbidities).

Abbreviations: n, number; OR, odds ratio; aOR, adjusted odds ratio; CI, confidence interval; Ref, reference group.

Sensitivity analysis applying the multivariable Cox proportional hazards regression model revealed aHR of mortality of 1.17 (95% CI, 1.01–1.35; P = 0.0371) for weekend vs. weekday admissions and 1.06 (95% CI 0.82–1.37; P = 0.6506) for holiday season vs. weekday admissions. Results of the sensitivity analysis were consistent with our main analyses, supporting the robustness of our findings.

### Analyses stratified by age, sex, type of aortic dissection, and hospital area

Stratified analyses revealed a similar trend of higher mortality risk for weekend admissions compared to weekday admissions, although most of these differences were not significant, likely due to insufficient statistical power after subgrouping of the study population (Table 4). Similarly, we did not find a statistically significant difference in mortality risk between the holiday season and weekday admission groups on analyses stratified by age, sex, aortic dissection type, or hospital area. The interaction tests demonstrated that none of the p for interaction reached statistical significance, supporting that our outcome estimates were not modified by difference of age, sex, aortic dissection type, and hospital area.

**Table 4:** Subgroup analyses for mortality risks among patients admitted for acute aortic dissection on the weekend compared to a weekday after stratification for age, sex, aortic dissection type, and hospital area.

	aOR*	95% CI	p-value	p for interaction
**In-hospital mortality**				
Age subgroup				0.9635
< 65 years	1.29	0.94–1.77	0.1149	
≥ 65 years	1.31	0.99–1.74	0.0580	
Sex subgroup				0.6641
Male	1.32	1.02–1.69	0.0317	
Female	1.25	0.86–1.82	0.2474	
Aortic dissection type				0.6117
Type A	1.50	0.68–3.29	0.3151	
Type B	1.27	1.02–1.59	0.0298	
Admission area				0.8684
North	1.26	0.91–1.76	0.1659	
Central	1.38	0.88–2.15	0.1637	
South	1.34	0.94–1.91	0.1069	
East	0.61	0.12–3.12	0.5494	
**7-day mortality**				
Age subgroup				0.3129
< 65 years	1.10	0.78–1.53	0.5951	
≥ 65 years	1.42	1.05–1.93	0.0234	
Sex subgroup				0.6058
Male	1.19	0.90–1.57	0.2214	
Female	1.42	0.97–2.09	0.0740	
Aortic dissection type				0.6485
Type A	1.36	0.60–3.05	0.4606	
Type B	1.23	0.98–1.56	0.0794	
Admission area				0.8386
North	1.40	0.98–2.00	0.0650	
Central	1.30	0.81–2.10	0.2787	
South	1.12	0.76–1.65	0.5553	
East	0.95	0.21–4.45	0.9525	
**180-day mortality**				
Age subgroup				0.5768
< 65 years	1.29	0.99–1.69	0.0617	
≥ 65 years	1.15	0.92–1.44	0.2199	
Sex subgroup				0.7057
Male	1.20	0.98–1.48	0.0800	
Female	1.14	0.83–1.55	0.4242	
Aortic dissection type				0.5360
Type A	1.36	0.70–2.64	0.0765	
Type B	1.17	0.98–1.40	0.3688	
Admission area				0.9187
North	1.24	0.94–1.63	0.1280	
Central	1.17	0.81–1.68	0.4148	
South	1.18	0.88–1.58	0.2594	
East	0.75	0.24–2.34	0.6255	

*The aOR is calculated by multivariable logistic regression model with adjustments for the characteristics listed in Table 1 (including age, sex, income level, aortic dissection type, hospital level, hospital area, Charlson comorbidity index score and comorbidities).

Abbreviations: aOR, adjusted odds ratio; CI, confidence interval.

## Discussion

We observed that an admission for acute aortic dissection on the weekend was associated with a higher in-hospital, 7-day, and 180-day mortality risk compared to admission on a weekday. A similar, but non-significant, association between admission during the holiday season and a higher mortality risk was identified.

Acute aortic dissection is a rare but catastrophic condition that is associated with a high rate of morbidity and mortality [14]. Effective management of this acute condition requires a team of emergency physicians, intensivists, cardiologists, cardiovascular surgeons, anesthesiologists, and nurses, with time to organize the care being critical. The barriers in organizing effective care on weekends has previously been described [5, 15], resulting in a “weekend effect” of higher risk of mortality [5]. These findings are similar to those of Qiu et al. who reported a higher rate of mortality among patients with an acute type A aortic dissection who underwent surgical intervention at night compared to the daytime [16].

In our study, patients with an acute aortic dissection who were admitted on the weekend had a 29% relatively higher risk of in-hospital mortality than those admitted on a weekday (14.3% vs. 11.5%, respectively). This finding is consistent with those of previous studies reporting worse outcomes for weekend admissions [5, 15]. We also identified a higher risk of 7-day and 180-day mortality for weekend compared to weekday admissions. These findings underline that the initial management of the patient might be more critical than follow-up efforts. We note that these short- and long-term mortality outcomes did not differ significantly between weekday and holiday season admissions. This finding was somewhat surprising and might be explained by insufficient statistical power due to the smaller sample size of the holiday season admission group.

Several factors are likely to contribute to the “weekend effect”. First, the staffing level might be relatively insufficient on weekends to deal with critical situations and emergency surgeries [17, 18]. Second, the relative workload per professional on the weekend is likely to be heavier and, thus, the efficiency of healthcare services available may be lower [19–22]. Third is the possibility that weekend team members may have had less experience working together than weekday teams which, again, might lower the efficiency of available healthcare services [18]. Concerning the specialist service, the on-duty surgeon and staff may not be familiar with every equipment and operation. In a previous study, Lee et al. reported that extracorporeal cardiopulmonary resuscitation (ECPR) on weekends was associated with lower survival and higher complications [23]. This finding may reflect unfamiliarity with medical devices and delayed emergency support. Besides, delayed involvement of a senior surgeon or specialist during weekends may lead to poor outcomes [24]. Access to hospital care and individual care-seeking behaviors are the potential factors contributing to the “weekend effect” that are outside the healthcare system mentioned by previous studies. Reduced access to primary and social care for vulnerable people like frail elderly during weekends may lead to delayed treatment [25]. Batal H et al. and David P Phillips et al. demonstrated that admissions to emergency department drop on weekends and holidays and spike immediately thereafter [26, 27]. This could be because some patients may procrastinate seeking medical care during weekends and holidays. Such behavior may deteriorate patients’ condition and affect the disease prognosis. Importantly, these factors can lead to delayed diagnosis and management, resulting in poorer outcomes despite the best efforts of surgeons and intensivists. This “weekend effect” demonstrates the unstable quality of the healthcare delivery system in Taiwan, and likely globally, between weekdays and weekends. This has significant implications for health policy. Sufficient weekend staffing, including the availability of subspecialty expertise, are important to overcome this problem. Further research is warranted to completely clarify the factors contributing to the “weekend effect” to appropriately inform healthcare policies to ensure a consistent quality of care on weekdays, weekends, and holidays.

We performed our study on a relatively large cohort of patients with an acute aortic dissection for three reasons. First, as for ruptured aortic aneurysms, emergent intervention is necessary to avoid patient death. As such, these patients provide a good model to evaluate the “weekend effect” [28]. Second was the identified need for further evaluation of the “weekend effect” as only a small number of studies have been published on this effect, including only one meta-analysis with high heterogeneity [4], with finding remaining controversial. Third, the additional “holiday season effect” has not been previously evaluated and yet is important as the reduction of staff is likely to be greater than on weekends, which further underlines the inconsistency in healthcare services. In a previous study on stroke patients, Huang et al. identified that admission during a holiday season was associated with a higher mortality risk compared to weekend admissions [29].

The main strength of our study is that it is representative of a nationwide cohort with sufficient clinical information included. In addition, this is the first study, to our knowledge, to evaluate the long-term (180-day) mortality risk, as well as to evaluate the “holiday season effect” for patients with acute aortic dissection. Our findings will be useful to inform healthcare policy to improve patient outcomes, regardless of the time and day of admission. The limitations of our study must also be acknowledged. First, as with other studies using data from the NHIRD, we could not obtain some detailed information, such as clinical details of the initial presentation, or laboratory and imaging reports [29]. Therefore, disease severity could not be controlled for among-group analyses. Besides, owing to the lack of detailed imaging reports, utilizing procedure codes and hospitalized surgery codes to distinguish type A from type B aortic dissection may be imprecise and could underestimate the proportion of type A aortic dissection. Further, we could not obtain information about medical equipment, surgeon expertise, and factors related to patient access to a hospital (e.g., interval from symptoms onset to treatment), which may also influence patients’ prognoses; thus, bias may have been introduced in our estimates, and causal relationship cannot be confirmed. Future studies are needed to address these issues. Second, our study population mainly comprised Taiwanese individuals; hence, our results may not be generalizable to other populations. Third, using the claims-based data, we could not determine if the patients had contact with other surgeons/experts at any other critical time in addition to the admission date (which we used to classify the exposure groups). This factor is a potential confounder and may dilute the effect that we observed. Finally, the sample size of the analysis for holiday season was relatively small, which caused the wide confidence interval and low statistical power. Such reasons may lead to the non-significant results in our analyses for holiday season vs. weekday. Given the difficulty collecting sufficient sample size in a single study to evaluate holiday season effect specifically on acute aortic dissection patients, additional studies and perhaps meta-analyses are warranted in the future.

## Conclusions

Patients admitted for acute aortic dissection during weekends are at higher risks of in-hospital, 7-day, and 180-day death compared to those admitted on weekdays. A significant association between holiday season admission and mortality was not observed. Based on our findings and current evidence, there might be a need to more carefully address medical staffing, including specialists, to provide effective healthcare services to optimize patient outcomes, regardless of the day and time of admission of acute aortic dissection.
